# Porcine Circovirus Type 4 Strains Circulating in China Are Relatively Stable and Have Higher Homology with Mink Circovirus than Other Porcine Circovirus Types

**DOI:** 10.3390/ijms23063288

**Published:** 2022-03-18

**Authors:** Xue Li, Si Chen, Guyu Niu, Xinwei Zhang, Weilong Ji, Ying Ren, Liying Zhang, Linzhu Ren

**Affiliations:** 1 College of Animal Sciences, Key Lab for Zoonoses Research, Ministry of Education, Jilin University, 5333 Xi’an Road, Changchun 130062, China; lixue9915@mails.jlu.edu.cn (X.L.); sichen20@mails.jlu.edu.cn (S.C.); niugy9916@mails.jlu.edu.cn (G.N.); xwzhang17@mails.jlu.edu.cn (X.Z.); jiwl19@mails.jlu.edu.cn (W.J.); 2 Public Computer Education and Research Center, Jilin University, 5333 Xi’an Road, Changchun 130062, China; ren_ying@jlu.edu.cn

**Keywords:** porcine circovirus type 4 (PCV4), three-dimensional (3D) structures modeling, epitope, homology, mink circovirus (MiCV), evolution

## Abstract

Porcine circovirus type 4 (PCV4) is a newly identified porcine circovirus (PCV) belonging to the *Circovirus* genus *Circoviridae* family. Although several groups have conducted epidemiological investigations on PCV4 and found that PCV4 also exists widely in pigs, there are few reports on the origin and evolution of PCV4. In this study, the genetic relationship between PCV4, mink circovirus (MiCV), bat circovirus (BtCV), PCV1, PCV2, and PCV3 was analyzed, and the consistency of viral proteins in three-dimensional (3D) structure and epitopes was predicted. We found that the genome and protein structure of PCV4 was relatively stable among current circulating PCV4 strains. Furthermore, PCV4 was more similar to MiCV in terms of its genome, protein structure, and epitope levels than other PCVs and BtCVs, suggesting that PCV4 may be derived from MiCV or have a common origin with MiCV, or mink may be an intermediate host of PCV4, which may pose a great threat to other animals and/or even human beings. Therefore, it is necessary to continuously monitor the infection and variation of PCV4, analyze the host spectrum of PCV4, and establish the prevention and treatment methods of PCV4 infection in advance.

## 1. Introduction

Circoviruses (CVs) containing the circular single-stranded DNA genome are widely distributed in bats, domestic and wild animals, humans, birds, and ticks. According to the classification of the international committee on the taxonomy of viruses (ICTV), forty-nine species of circoviruses in the *Circovirus* genus *Circoviridae* family have been identified so far. Among circoviruses, the most reported and economically important viruses are porcine circoviruses (PCVs).

PCVs are viruses that mainly infect pigs, which include at least four species, PCV1, PCV2, PCV3, and PCV4, to date. PCV1 was first identified in porcine kidney cell line 15 (PK-15) and was considered a contaminant of the cells due to its non-pathogenicity. PCV2 infection is widespread in pig farms all over the world, causing porcine circovirus diseases (PCVD), which are usually co-infected with other pig pathogens, resulting in a secondary infection [1,2]. Apart from pigs, PCV2 can also infect mice and cattle [1], and even human cell lines [3]. PCV3 was first discovered in pigs with porcine dermatitis and nephropathy syndrome (PDNS)-like clinical signs in 2016 in the USA; since then, the number of PCV3 infections in pig farms around the world has increased [4]. PCV3 has a broad-spectrum host, including pig, wild boar, ticks, dog, cattle, and mice [4]. The most recently reported PCV, namely, PCV4, was first identified in Chinese farmed pigs with severe clinical disease in 2019 [5]. Thereafter, several groups have conducted epidemiological investigations on PCV4 and found that PCV4 also exists widely in pigs [6,7,8,9,10,11,12]. Kim et al. found that the positive rates of PCV4 in individual pig samples and at the farm level were 39.3% and 45.7%, respectively [9]. The virus can be detected in all age groups and aborted fetuses, with the highest titer in the sow group [8]. However, there are few reports on the origin and evolution of PCV4. In the present study, the genetic relationship between PCV4, mink circovirus (MiCV), bat circovirus (BtCV), PCV1, PCV2, and PCV3 was analyzed, and the consistency of viral proteins in three-dimensional (3D) structure and epitopes was predicted.

## 2. Results

### 2.1. The Genome and Protein Structure of PCV4 Strains Circulating in China Are Relatively Stable

Up to now, only twenty-eight complete genomic sequences of PCV4 were submitted to NCBI, including twenty-seven sequences from China and one from South Korea (Supplemental Table S1). The complete genome of PCV4 is about 1770 nucleotides (nt) in length with a circular single-stranded genomic DNA, encoding two main open reading frames (ORFs) [5]. Like PCV1, PCV2, and PCV3, the ORF1 of PCV4 encodes replicase (Rep), and the ORF2 encodes Capsid (Cap) [5]. Therefore, twenty-eight sequences of PCV4 obtained from NCBI were analyzed in the following study.

**Figure 1:**
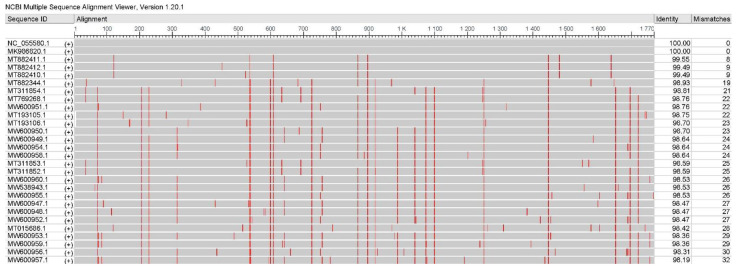
Multiple alignments of PCV4 strains. A full-length sequence of PCV4 HNU-AHG1-2019 (GenBank No. NC_055580.1) was used as the reference sequence, and the multiple alignments were conducted and visualized using online software NCBI Multiple Sequence Alignment Viewer (version 1.20.1).

First, the complete sequences of PCV4 were aligned with the reference sequence HNU-AHG1-2019 (GenBank No.: NC_055580.1) via online software NCBI Multiple Sequence Alignment Viewer (version 1.20.1, NCBI, MD, USA). As shown in Figure 1, a total of 26 major mutation sites were identified in 27 strains compared with that of the reference strain. The identity rates of the indicated sequence and the reference sequence were 98.19% to 99.55%, and the number of mismatches between the indicated sequence and the reference sequence ranged from 8 nt to 32 nt. Furthermore, most of the mutations were synonymous, while 6 mutations were non-synonymous mutations, including G84A, C537A, G757A, T1100G, T1448C, and T1654C (Table 1). The first three mutations (G84A, C537A, and G757A) were in the viral Rep protein, and the latter three (T1100G, T1448C, and T1654C) occurred in the viral Cap protein. The mutated amino acids H228, L212, V93, and S27 are similar in structure to the original amino acids R228, M212, I93, and N27, which have little or no effect on the structure of the protein. Notably, two mutations located in the Rep protein, A4T and Q155K, are different from the original amino acids in structure. The A4T mutation is a mutation from nonpolar, aliphatic amino acid (alanine) to polar, hydrophilic, uncharged amino acid (threonine), and the Q155K mutation is a mutation from charge-neutral, polar amino acid (Glutamine) to charged, hydrophilic, aliphatic amino acid (Lysine). The strong charge ensures lysine is easily located on the outer hydrophilic surface of virus protein, while the flexibility of the long side chain with a positively charged end makes lysine suitable for binding with negatively charged molecules on its surface, such as DNA.

**Figure 2:**
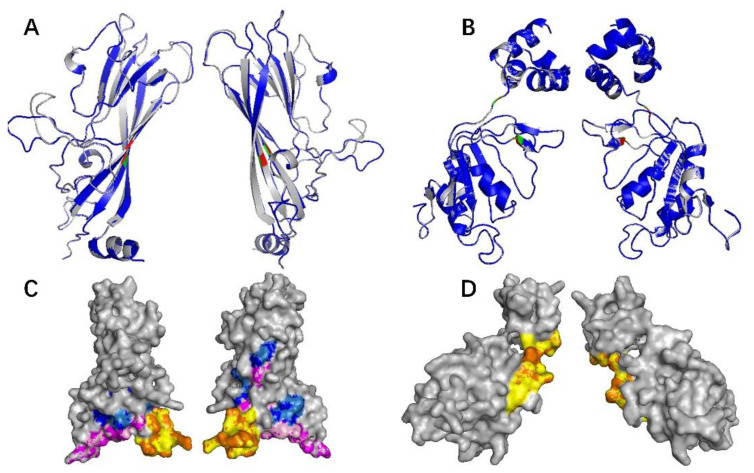
Analysis of structure and antigen epitopes of PCV4 Cap and Rep proteins. (**A**,**B**) 3D structure alignment of mutated and reference PCV4 Cap (**A**) and Rep (**B**). 3D Structure of the PCV4 Cap was generated via homology modeling using bat circovirus Cap (PDB ID: 6RPO) as a template. Gray indicates the reference PCV4 Cap, and blue means the mutated PCV4 Cap. Amino acids in the reference Cap and Rep were labeled red, while mutations were labeled green. RMSD value of PCV4 mutant and original capsid was 0.045 Å, while the RMSD of PCV4 Reps was 0.002 Å. (**C**,**D**) Predicted antigen epitopes of mutated and reference PCV4 Cap (**C**) and Rep (**D**). The predicted epitopes were indicated by various colors. Epitopes 1, 2, and 3 of reference PCV4 Cap were labeled by orange, light pink, and sky-blue, respectively. Epitopes 1, 2, and 3 of mutated PCV4 Cap were labeled by yellow, purple, and blue, respectively. Epitope 1 of reference PCV4 Rep was labeled by orange, while epitope 1 of mutated PCV4 Rep was labeled by yellow.

To further evaluate the effect of the mutations on the structure of the protein, protein homology modeling was performed by comparing the secondary structure of mutant protein and original protein. As shown in Figure 2A, the mutations I93V and M212L in the secondary structure of viral Cap protein were in β-fold, whereas N27S is located in α helix. The mutations A4T and Q155K in the secondary structure of viral Rep protein are located in random curl, while R228H is located in β-fold (Figure 2B). However, the 3D structures of the PCV4 Rep proteins (Figure 2A) and Cap proteins (Figure 2B) are almost the same as the Root Mean Square Deviation (RMSD) values for the structural alignment of viral capsid between PCV4 mutant and the original strain was 0.045 Å, while the RMSD values for PCV4 Reps of two strains was 0.002 Å, which indicates that the structure of the mutant protein was quite similar to that of the original protein, and mutation has no effect on the structure of viral proteins. This may be because the polarity and acidity and alkalinity of the mutated amino acid are consistent with those of the original amino acid, which has little influence on the protein conformation.

**Figure 3:**
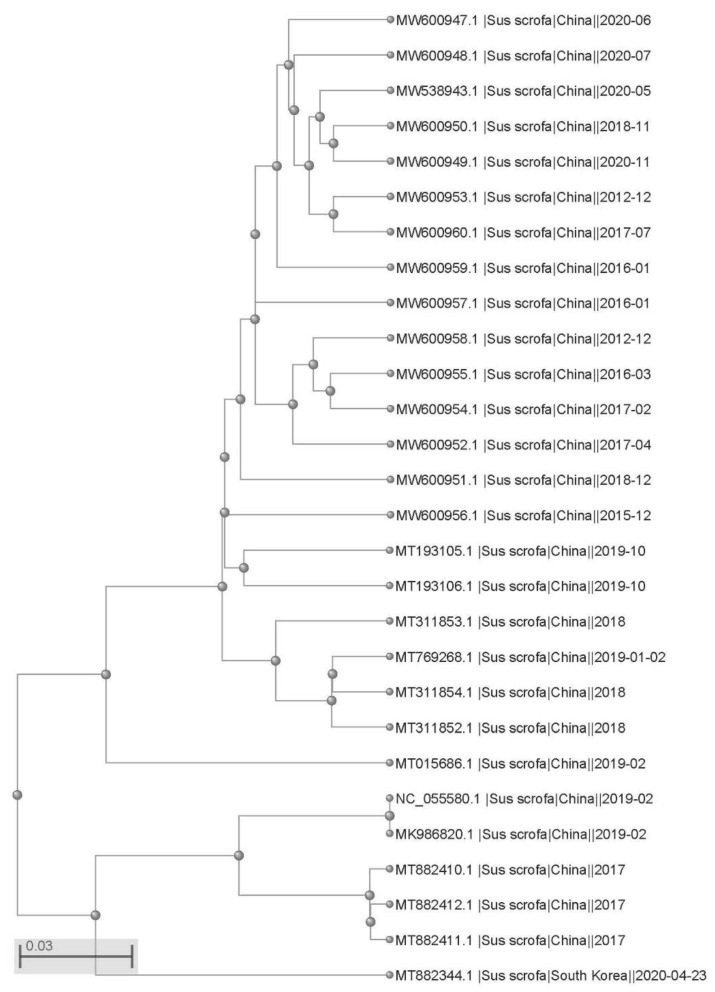
Phylogenetic tree of PCV4 strains. NCBI Tree Viewer JS version 1.19.2 was used to build the phylogenetic tree.

The above results were further confirmed by the prediction of B-cell epitopes from antigenic sequences using the ABCpred dataset and Pymol program. The epitopes of N27S, I93V, and M212L mutants of Cap protein were found to be consistent with the 3D structure of the original epitopes (Figure 2C), while Q155K mutation of Rep protein did not change the 3D structure of the epitope (Figure 2D), which proved that amino acid substitution in Cap and Rep protein of PCV4 did not affect its antigenicity. Moreover, phylogenetic analysis showed that 28 PCV4 strains can be divided into two clades (Figure 3), which is consistent with the results reported by other groups previously [13,14].

These results demonstrate that the polarity, acidity, and alkalinity of the mutated amino acids were consistent with those of the original amino acids, which had little effect on the conformation of the protein. The existing PCV4 mutation did not affect its immunogenicity, and the PCV4 strain was stable. However, it is necessary to continuously monitor the prevalence and variation of PCV4.

### 2.2. PCV4 Has Higher Homology with MiCV Than Other PCVs

**Figure 4:**
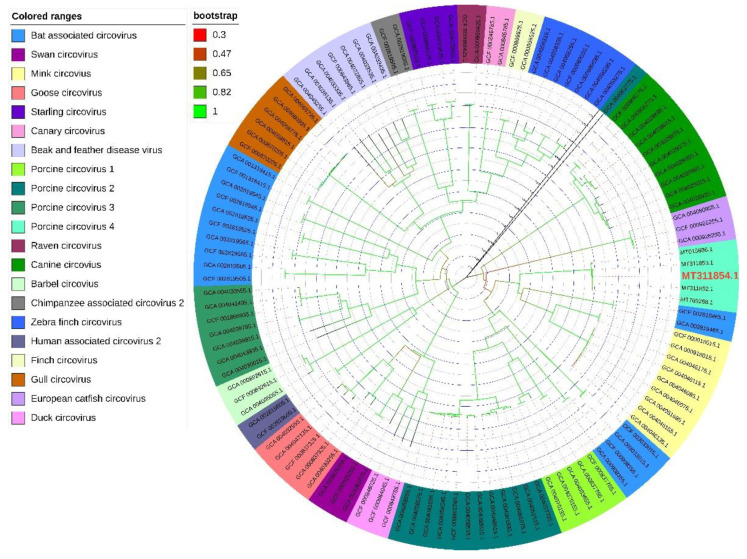
Phylogenetic tree of PCV4 and other circoviruses. The homology tree based on the whole genome sequences of the above circoviruses was analyzed using the Neighbor-Joining (NJ) method in Mega 6.0 software.

Five sequences of PCV4 strains were used to construct a homology tree with available sequences of other circoviruses. As shown in Figure 4, PCV4 belongs to a branch with BtCV and MiCV, suggesting these viruses have high homology. Furthermore, PCV1, PCV2, and PCV3 were divided into different evolutionary branches from PCV4.

**Figure 5:**
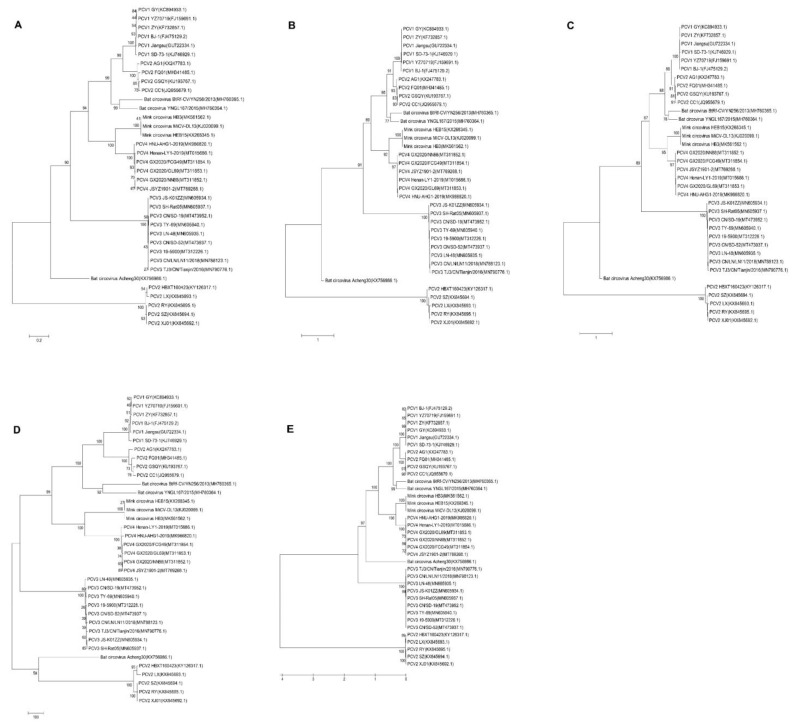
Phylogenetic tree based on the whole genomic sequences of BtCV, MiCV, and PCVs. (**A**) Maximum Likelihood (ML). (**B**) Neighbor-Joining (NJ). (**C**) Minimum Evolution (ME). (**D**) Maximum Parsimony (MP). (**E**) The unweighted pair-group method with arithmetic mean (UPGMA). The homology trees based on the whole genomic sequences of the circoviruses were analyzed using the indicated method in Mega 6.0.

Moreover, five evolutionary trees based on the whole genome sequences of BtCV, MiCV, and PCVs were reconstructed using five different phylogenetic methods. As shown in Figure 5, the phylogenetic trees constructed by five methods were similar, indicating genotyping based on complete coding sequences of PCV4 is stable and accurate, and PCVs and MiCV may originate from BtCV. PCV1, PCV3, and PCV4 have little variation, and there is no obvious lineage change among the strains. However, PCV2 varies greatly in the process of evolution, forming two branches. The first branch (PCV2 HBXT160423, SZ, LX, RY, and XJ01) is relatively old, and has a close genetic relationship with the BtCV strain (GenBank No.: KX756986.1). The other branch (PCV2 AG1, FQ01, GSQY, and CC1) is closely related to PCV1 (GenBank Nos.: MH760365 and MH760364) and BtCV. Furthermore, PCV2 may appear before PCV1, and PCV4 has higher homology with MiCV than other PCVs.

**Figure 6:**
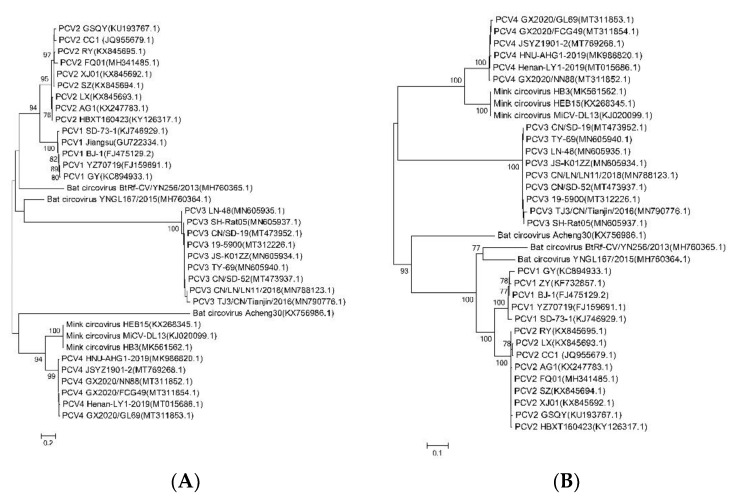
Phylogenetic tree based on the amino acids sequences of BtCV, MiCV, and PCVs. The homology trees based on the amino acids sequences of Cap (**A**) or Rep (**B**) of the circoviruses were analyzed using the Neighbor-Joining (NJ) method in Mega 6.0.

Meanwhile, PCV4 and MiCV were grouped into the same branch in five different phylogenetic trees based on the whole genomic sequences (Figure 5). These results were further confirmed by phylogenetic analysis based on the amino acids sequences of BtCV, MiCV, and PCVs (Figure 6).

Moreover, as shown in Table 2, the genomes, *Rep* and *Cap* genes of PCV1 and PCV2, have the highest homology (50.1–75.9%) with the corresponding sequences of BtCV. However, the *Rep* and *Cap* genes of PCV1 and PCV2 showed higher similarity to different BtCV sequences. The *Rep* and *Cap* genes of PCV3 showed higher identity to MiCV and BtCV sequences, respectively. Notably, the genomes, *Rep* and *Cap* genes of PCV4, showed the highest homology to the MiCV sequences.

These results indicate that the homology between PCV4 and MiCV is higher than that of BtCV, suggesting PCV4 may be derived from MiCV or have a common origin with MiCV.

### 2.3. The 3D Structure of Cap Protein of PCV4 Was Most Like That of MiCV

**Figure 7:**
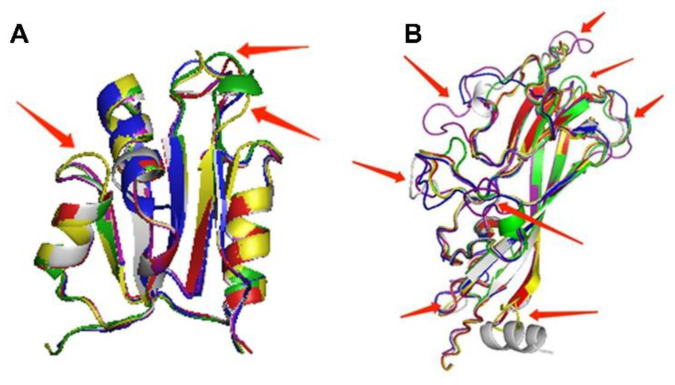
Comparison of 3D structures of Rep and Cap proteins of PCVs, BtCV, and MiCV. PCV1, red. PCV2, yellow. PCV3, green. PCV4, blue. MiCV, white gray. BtCV, purple. (**A**) Rep. (**B**) Cap.

To further confirm the above results, the 3D structure of viral Cap and Rep were compared. The 3D structures of Rep proteins of BtCV, MiCV, and PCVs are highly consistent, and the difference is mainly located in the loop region (Figure 7A). The 3D structure of Rep in different PCVs was not much different, but the 3D structure of Rep protein in PCV2 and PCV3 was similar, while that in PCV3 and PCV4 was quite different. The 3D structure of PCV1 Rep was most similar to that of BtCV and MiCV Rep proteins.

The 3D structure of Cap proteins of different circoviruses was quite different from that of Rep proteins (Figure 7B), which is consistent with the results of homology comparison between genomes and *Cap* genes mentioned above (Table 2). However, the α-helix and β-fold of the 3D structure of Cap proteins were consistent, and the differences were mostly located in the loop region. The 3D structure of Cap in different PCVs was not much different, but the 3D structure of Cap protein in PCV1 and PCV2 was similar. The 3D structure of Cap protein of PCV3 was quite different from that of other circoviruses, and the 3D structure of Cap protein of PCV3 was the most different from that of MiCV and BtCV. Furthermore, the 3D structure of Cap protein of PCV4 was most similar to that of MiCV, which is consistent with the homology of the genome.

### 2.4. The Epitopes of PCV4 Cap Were Most Like That of MiCV

**Figure 8:**
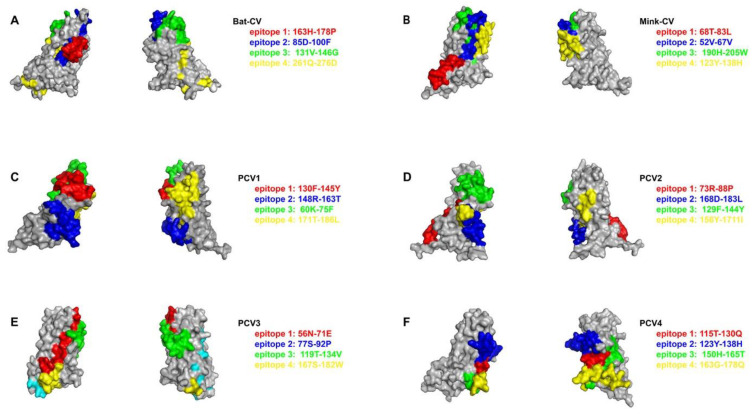
Predicated antigen epitopes of Cap of circoviruses. (**A**) Bat circovirus. (**B**) MiCV. (**C**) PCV1. (**D**) PCV2. (**E**) PCV3. (**F**) PCV4. Antigen epitopes with the same score are represented by the same color.

As Cap protein is closely related to the antigenicity and/or pathogenicity of circoviruses, the antigenic epitopes of Cap proteins of BtCV, MiCV, PCV1, PCV2, PCV3, and PCV4 were predicted using ABCpred and Pymol online software. Figure 8 showed B cell epitopes with higher scores in Cap proteins of circoviruses from the bat, mink, and pigs. Although the 3D structures of Cap proteins were different, the epitopes of different Cap proteins of circoviruses were mostly located in the loop region. Moreover, regarding Cap protein, the RMSD value of MiCV and PCV4 was 0.336 Å, which was lower than that of the BtCV (1.170 Å), PCV1 (0.674 Å), PCV2 (0.720 Å), and PCV3 (2.706 Å), indicating that the structure of PCV4 Cap was more similar to that of MiCV than other CVs. However, the RMSD value of Rep protein between PCV4 and BtCV, as well as MiCV, was 0.304 Å and 0.357 Å, respectively, indicating the structure of PCV4 Rep was more like that of BtCV than that of the MiCV.

**Figure 9:**
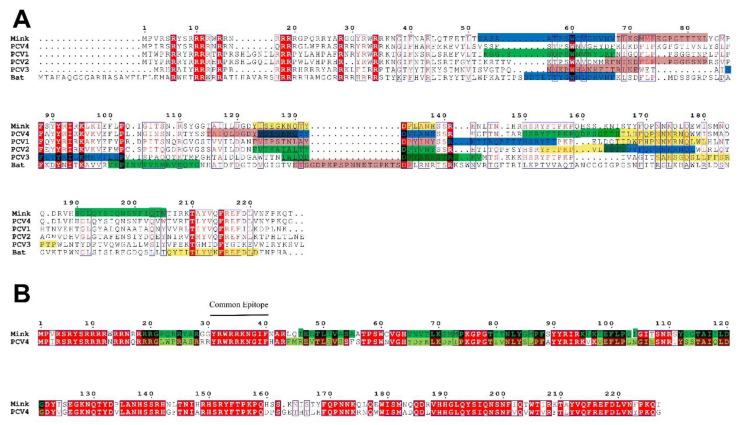
Multiple alignments of Capsid sequences of circoviruses. Conservative amino acid sites are marked in red, and conservative regions are marked in blue boxes. (**A**) Amino acid sequence alignment and potential epitope of CVs Cap protein. Based on the prediction of epitope scores, four potential epitopes in the 3D structure of CVs Cap protein were labeled with pink, blue, green, and yellow in turn. (**B**) Comparison of amino acid sequences and potential epitopes of Cap protein between PCV4 and MiCV. The epitope of MiCV Cap protein is green, and that of PCV4 Cap protein is yellow-green.

To clarify the homology of different CVs Cap proteins, the amino acid sequences of Cap proteins were compared by the Clustal W method, and it was found that there were some conserved amino acid sites (R6, R10, R11, R14, R18, R19, R28, R34, W60, F88, Y91, I93, K95, P102, R141, T210, and F215) and semi-conserved regions among the Cap proteins of these strains (Figure 9A). Furthermore, the amino acid sequence identity of Cap proteins of PCV4 and MiCV was as high as 78.3%, but the sequence identity of PCV4 with other strains was relatively low (BtCV: 18.6%; PCV1: 47.7%; PCV2: 48%; and PCV3: 27.6%). Moreover, to determine whether the inconsistency of CVs Cap sequence homology will lead to the inconsistency of CVs Cap protein epitopes, and ABCpred was used to predict the common epitopes of CVs or the epitopes with the greatest differences. The results showed that PCV4 and MiCV were highly consistent in the epitopes of CVs Cap protein, which was consistent with sequence homology (Figure 9A,B), suggesting that PCV4 was closer to MiCV. Notably, there was a common epitope (Y^31^RWRRKNGIF^40^) in Cap sequences of PCV4 and MiCV, which may be the target of cross-protection between the two CVs. Therefore, PCV4 has higher homology, especially epitopic similarity, with MiCV than other PCVs.

## 3. Discussion

Up to now, four types of PCVs have been discovered in pigs; however, the Cap proteins of different types of PCVs are quite different. In the present study, the genetic relationship between PCV4 and MiCV, BtCV, PCV1, PCV2, and PCV3 was analyzed, and the consistency of Cap proteins in 3D structure and epitopes was predicted. The results showed that PCV4, as a newly identified virus, has a higher affinity (about 70–80% at maximum) with MiCV than other PCVs, suggesting that PCV4 may be derived from MiCV or have a common origin with MiCV. Notably, the host spectrum of PCV4 is still unknown, but whether PCV4 can infect other animals or even humans needs further evaluation. We previously found that PCV2 can infect and replicate in human cells [3], and PCV3 can infect several hosts, including domestic and wild pigs, dogs, cattle, and ticks. [4]. Furthermore, the RMSD value of MiCV Cap and PCV4 Cap was the lowest, while the RMSD value of BtCV Rep and PCV4 Rep was lower than that of other CVs, indicating that the structure of PCV4 Cap was similar to MiCV, while that of PCV4 Rep was similar to that of BtCV. Besides, we previously found that MiCV can infect several animals, including mink, fox, and raccoon dog [15]. Fox is the most sensitive to MiCV, followed by mink and raccoon dog [15]. Therefore, whether mink or other animals act as an intermediate host for recombination, variation, and cross-species transmission of PCV4 or other circoviruses remains to be clarified, and it is necessary to pay more attention to the biosafety of economic animals such as mink, fox, and raccoon dog.

Moreover, PCV2 has been identified for decades, and it is divided into at least eight subclades based on the *Cap* gene, including PCV2 2a to 2h [16]. To date, PCV2a, PCV2b, and PCV2d are the dominant genotypes in the field. We also found that PCV1, PCV3, and PCV4 have little variation, and there is no obvious lineage change among the strains (Figure 5). However, PCV2 formed two branches based on the whole genomic sequences (Figure 5) but was grouped into the same branch based on the amino acids sequences (Figure 6). The possible reason was that synonymous mutations in the genome affected the phylogenetic analysis based on the genome, not protein. The homology of *Cap* genes of different PCV2 strains is consistent with that of the whole genome, and the difference of the *Cap* genes may determine the genetic distance between different strains. Furthermore, it was found that the mutation rates of PCV3 and PCV4 viruses were low [7,17,18]. We also found in this study that PCV4 strains had a high degree of genetic stability. However, like other viruses, the evolutionary adaptation of PCV4 may promote the mutation of PCV4 and generate a pathogenic strain in pigs or other intermediate hosts. There are many reports about the pathogenicity of PCV2 and PCV3 [1,2,19,20,21,22], but few reports were about the pathogenicity of PCV4 to date, partly due to the failure of isolation of PCV4. Therefore, it is necessary to closely monitor the variation of PCV4, find out the cross-species transmission of PCV4 in time, and avoid the occurrence of an epidemic. In addition, we recently successfully rescued PCV4 from an infectious clone [23]. The rescued PCV4 can be replicated and propagated in PK-15 cells and piglets, which can be detected in almost all samples collected from the challenged piglets [23]. Although there were no obvious clinical symptoms in piglets inoculated with the rescued PCV4, visible pathological changes were found in several organs of piglets inoculated with the rescued PCV4, which indicated that the rescued PCV4 was pathogenic in piglets [23]. Furthermore, viremia, PCV4-specific antibodies, and up-regulated cytokines/chemokines were also detected in the group inoculated with PCV4, indicating that effective humoral and cellular immune responses were stimulated in response to the virus inoculation [23]. The study of the pathogenesis of PCV4 and related works is still in progress.

Moreover, because Cap protein is an important structural protein of CVs, its conserved region and common epitope can be used as an important site for monitoring virus evolution and variation and may also become a potential target for developing multiple-valent or universal antibodies or drugs against CVs. This is still of great significance for the prevention and treatment of circovirus-related diseases.

## 4. Materials and Methods

### 4.1. Multiple Alignments

To evaluate the genetic variation of PCV4 epidemic strains in China, 28 complete sequences of PCV4 were collected from GenBank (Supplemental Table S1), followed by multiple alignments and epitope prediction. A full-length sequence of PCV4 strain HNU-AHG1-2019 (GenBank No. NC_055580.1) was used as the reference sequence, and the multiple alignments were conducted and visualized using the online program NCBI Multiple Sequence Alignment Viewer (version 1.20.1, NCBI, MD, USA).

To compare the relationship between PCVs, bat circovirus (BtCV), and mink circovirus (MiCV), the full-length genome, Cap, and Rep protein sequences of BtCV, MiCV, and PCVs were used as templates (Supplemental Table S2). The sequence alignments were performed using ClustalW of Mega version 6 [24].

### 4.2. Phylogenetic Tree

A phylogenetic tree of PCV4 strains was built using NCBI Tree Viewer JS version 1.19.2 (NCBI, MD, USA).

To elucidate the relationship between PCVs, BtCV, and MiCV, the phylogenetic tree of the full-length genome nucleotide sequences of different circoviruses was constructed. Phylogenetic analysis was performed using Maximum Likelihood (ML) [25], Neighbor-Joining (NJ) [26], Minimum Evolution (ME) [27], Maximum Parsimony (MP), and unweighted pair-group method with arithmetic mean (UPGMA) in MEGA6 [28] with a bootstrap of 1000 replicates [28,29].

### 4.3. Three-Dimensional (3D) Structures Modeling

Three-dimensional (3D) structures of Cap proteins of PCV1 (GenBank No.: FJ475129.2) and PCV2 (GenBank No.: JQ955679.1) were constructed based on the 3D structure of PCV2 Cap (Protein Data Bank (PDB) ID: 6E2R) using the online program Swiss-Model (https://swissmodel.expasy.org/ (accessed on 13 March 2022) according to the protocol described in the program [30].

The 3D structures of Cap proteins of PCV3 (GenBank No.: MN790776.1), PCV4 (GenBank No.: MT311854.1), BtCV (GenBank No.: KX756986.1), and MiCV (GenBank No.: KJ020099.1) were constructed based on the 3D structure of BtCV Cap (PDB ID: 6RPO). The 3D structures of Rep proteins of PCVs, BtCV, and MiCV were generated based on the 3D structure of PCV2 Rep (PDB ID: 5XOR).

The 3D structural alignment was performed by the online program Pymol 2.0. RMSD (Schrödinger, NY, USA) analysis for the structural alignment of viral proteins was conducted according to the protocol described by Souza et al. [31].

### 4.4. Epitope Prediction

Epitope prediction was performed to evaluate B cell epitopes of Cap proteins from different circoviruses using the online server ABCpred (www.imtech.res.in/raghava/abcpred/, accessed on 13 March 2022) [32]. The threshold was set to 0.5. The length of B cell epitopes was set to 10 to 20 amino acids (aa), respectively.

The predicted epitopes of Cap proteins with high scores were evaluated and analyzed using the online program Pymol 2.0, followed by RMSD analysis.

## 5. Conclusions

In this study, we found that the genome and protein structure of PCV4 was relatively stable among current circulating PCV4 strains. Furthermore, PCV4 was more similar to MiCV in the genome, protein structure, and epitope level than other PCVs and BtCVs, suggesting that PCV4 may be derived from MiCV or have a common origin with MiCV, or mink may be an intermediate host of PCV4. Therefore, it is necessary to continuously monitor the infection and variation of PCV4, analyze the host spectrum of PCV4, and establish the prevention and treatment methods of PCV4 infection in advance.

## Figures and Tables

**Table 1:** Major mutations in PCV4 strains.

Region (Location)	Genomic Site	Mutation	
		Nucleotide Acid	Amino Acid
UTR (1–74)	72	C-T	synonymous
	74	G-A	synonymous
Rep (75–965)	84	G-A	A4T
	206	C-T	synonymous
	228	T-C	synonymous
	314	C-T	synonymous
	536	C-G/T	synonymous
	537	C-A	Q155K
	599	T-C	synonymous
	608	A-C	synonymous
	641	A-G	synonymous
	726	C-T	synonymous
	752	T-C	synonymous
	757	G-A	R228H
	866	C-T	synonymous
	896	C-T	synonymous
	920	G-A	synonymous
UTR (966–1046)	988	C-T	synonymous
	1040	C-T	synonymous
Cap (complement, 1047–1733)	1074	A-G	synonymous
	1100	T-G	M212L
	1251	T-G	synonymous
	1448	T-C	I93V
	1654	T-C	N27S
	1698	C-A	synonymous
	1722	T-C	synonymous

**Table 2:** Sequence homology of BtCV, MiCV, and PCVs. The bold means highlights for the contents.

Species	GenBank No.	PCV1 (%)			PCV2 (%)			PCV3 (%)			PCV4 (%)		
		Genome	*Rep* Gene	*Cap* Gene	Genome	*Rep* Gene	*Cap* Gene	Genome	*Rep* Gene	*Cap* Gene	Genome	*Rep* Gene	*Cap* Gene
Bat	KX756986	23.6~24.5	52.7~54.4	23.6~24.5	26.8~26.0	53.2~54.2	26.8~26.0	17.0~17.5	46.0~46.4	17.0~17.5	28.5~29.0	52.8~53.1	28.5~29.0
	MH760364	**50.2~51.5**	69.4~70.7	**50.2~51.5**	**50.7~53.5**	71.2~71.9	**50.7~53.5**	**28.8~29.3**	46.0~46.7	**28.8~29.3**	45.7~46.2	47.4~48.1	45.7~46.2
	MH760365	47.2~48.9	**73.9~75.9**	47.2~48.9	46.1~47.2	**73.4~74.0**	46.1~47.2	22.5~24.0	45.0~45.7	22.5~24.0	43.5~44.4	50.5~51.2	43.5~44.4
Mink	KJ020099	45.2~45.7	50.2~51.4	45.2~45.7	44.3~45.0	49.3~50.0	44.3~45.0	24.9~25.4	**49.7~50.0**	24.9~25.4	**69.6~70.0**	**80.1~80.7**	**69.6~70.0**

## Data Availability

All data generated or analyzed during this study are included in this published article and its supplementary information files.

## Supplementary Materials

The following supporting information can be downloaded at: https://www.mdpi.com/article/10.3390/ijms23063288/s1.
